# Treatment Fidelity during Therapist Initial Training is related to Subsequent Effectiveness of Parent Management Training—Oregon Model

**DOI:** 10.1007/s10826-017-0706-8

**Published:** 2017-03-29

**Authors:** Jill Thijssen, Gonnie Albrecht, Peter Muris, Corine de Ruiter

**Affiliations:** 1 Department of Clinical Psychological Science, Maastricht University, Maastricht, The Netherlands; 2 Department of Developmental Psychology, Tilburg University, Tilburg, The Netherlands; 3 PI Research, Duivendrecht, The Netherlands

**Keywords:** Parent Management Training - Oregon Model (PMTO), Treatment fidelity, Treatment outcome, Working alliance, Treatment completion

## Abstract

The present study examined the association between treatment fidelity during therapist initial training and subsequent treatment outcome of Parent Management Training - Oregon model (PMTO) in The Netherlands. Clinically referred children (*N* = 86) aged 4 to 11 years and their parents received PMTO and were assessed at four time points: at baseline, and after 6, 12, and 18 months. Difference scores between baseline and follow-up assessments of externalizing behavior problems, parenting practices, and parental psychopathology and parents' overall ratings of working alliance, were correlated with treatment fidelity scores measured prior to the intervention. Furthermore, differences in therapists' fidelity scores between treatment completers and drop-outs were examined. Results showed that higher fidelity scores of PMTO therapists during initial training were associated with larger improvements in externalizing behavior, parenting practices, and parental psychopathology, especially after 18 months. In addition, parents who completed the treatment had a significantly more adherent therapist than families who dropped out. However, the correlations between treatment fidelity and working alliance were non-significant. These findings indicate that therapists' high adherence to the PMTO treatment principles during initial training decreases the chance of treatment drop-out and positively affects the longterm effectiveness of PMTO.

## Introduction

Several longitudinal studies have demonstrated that early childhood behavioral problems such as disobedience, fighting, lying, and stealing are predictive of antisocial conduct and delinquency in later life (Coté et al. 2006; Simonoff et al. 2004). Moreover, the earlier these externalizing problems begin, the higher the risk of serious problems during adulthood (Moffitt 1993; Patterson et al. 1989; Reef et al. 2011). The fact that externalizing behavior problems in children may have adverse long-term consequences underlines the importance of early intervention programs. Many of these programs focus on the improvement of parenting practices and there is indeed evidence showing that the enhancement of positive and more effective parenting strategies has positive effects on children’s development of prosocial behavior (DeGarmo and Forgatch 2005 2007; Forgatch and DeGarmo 1999; Martinez and Forgatch 2001; Ogden and Amlund-Hagen 2008).

Parent Management Training - Oregon model (PMTO) is a program especially developed for the parents of children between 4 and 12 years of age with severe externalizing behavior problems such as Oppositional Defiant Disorder or Conduct Disorder. PMTO is based on the Social Interaction Learning (SIL) model, which assumes that contextual factors, such as socio-economic disadvantage and parental psychopathology, have a negative impact on child outcomes by undermining parenting quality (Snyder and Patterson 1995). Therefore, PMTO focuses on teaching parents how to reduce negative, counterproductive parenting practices and to replace these with positive, more effective parenting practices, such as encouragement (i.e., stimulation of prosocial behaviors in the child, for example by using positive reinforcement), effective discipline (i.e., consistent use of mild sanctions), monitoring (i.e., keeping track of the child’s activities), problem solving (i.e., responding adequately to rule-breaking behaviors and settling arguments with the child), and positive involvement (i.e., giving love and warm attention to the child; Forgatch and DeGarmo 1999; Forgatch et al. 2005).

In previous studies, PMTO has been shown to produce positive effects on child externalizing behavior outcomes and parenting practices, and this appears not only true in the United States where the program was developed (e.g., Forgatch and DeGarmo 1999; Forgatch et al. 2009), but also in other countries such as Norway, Iceland, and The Netherlands (Ogden and Amlund-Hagen 2008; Sigmarsdóttir et al. 2014; Thijssen et al. 2017). However, the effectiveness of PMTO depends on the therapists’ treatment fidelity. High treatment fidelity is the delivery of the treatment according to the treatment principles performed with good clinical and teaching skills that promote behavior change (Forgatch et al. 2005). Prior research has provided tentative evidence indicating that therapists’ close adherence to the treatment protocol is indeed associated with greater improvements in parenting skills and more clear-cut reductions of externalizing behavior problems, and this appeared not only true for PMTO (Forgatch and DeGarmo 2011; Forgatch et al. 2005; Hukkelberg and Ogden 2013) but also for other interventions aimed at externalizing behavior problems in children (e.g., Hogue et al. 2008; Huey et al. 2000). These previous studies assessed treatment fidelity during the course of treatment. However, the goal of the present study is to evaluate whether fidelity ratings of PMTO therapists during initial training (i.e., at certification) are related to changes in parent and child behaviors subsequently receiving PMTO. In the present design, treatment fidelity is measured on the basis of therapy sessions used for therapists’ certification, and thus assessed entirely independent from treatment effectiveness, which is determined on subsequent treatments conducted by the therapists. Within PMTO, treatment fidelity is measured by using the Fidelity of Implementation (FIMP) rating system (Knutson et al. 2009) which evaluates the therapist on five dimensions: PMTO knowledge, Structure, Teaching, Process skills, and Overall development. The FIMP is a standard part of the PMTO training program to evaluate the progress of PMTO therapists and it is also used for certification and recertification.

The present study relies on the data collected for a study evaluating the effectiveness of PMTO in The Netherlands (Thijssen et al. 2017). In this study, only certified PMTO therapists were involved, which implies that all therapists had at least a sufficient FIMP score. However, therapists may still show considerable variation in the extent to which they adhere to the method, with some of them being really strict at following the guidelines of the intervention and others carrying out the protocol in a looser fashion. First, we will examine whether treatment fidelity scores as obtained during training (i.e., at certification) are associated with later treatment outcome in terms of externalizing behavior problems, parenting practices, parental psychopathology, and working alliance. Second, we will examine whether treatment fidelity was related to treatment completion. We expected that higher treatment fidelity scores of the therapists would be associated with larger improvements on outcome measures and higher rates of treatment completion. The current study’s procedure was different from previous studies on treatment fidelity in PMTO (Forgatch and DeGarmo 2011; Forgatch et al. 2005; Hukkelberg and Ogden 2013) in a number of ways: the present study (a) included multiple outcome measures of PMTO instead of only parenting practices or externalizing behavior; (b) examined the FIMP dimensions separately instead of using a mean or a single construct; and (c) examined the association between fidelity scores and treatment outcome at different assessment points. This new approach could provide new insights into the association between PMTO treatment fidelity and treatment outcomes.

## Method

### Participants

FIMP scores were available for 86 of the 91 families receiving PMTO. Children and their parents were recruited through five child service agencies across The Netherlands. The mean age of these children (62 were boys and 24 were girls) was 7.16 years (SD = 1.81). Based on the Diagnostic Interview Schedule for Children (DISC; Shaffer et al. 2000), 73.3% of the children met the DSM diagnostic criteria for ADHD, 65.6% for ODD, and 12.8% for CD. There were 9 children who did not meet the full criteria of these disorders. Children had a mean IQ of 100.87. Mean age of the main caregivers was 38.51 years (*SD* = 1.27); 25% of them were single parents and 76% had the Dutch nationality. Approximately 44% had an Intermediate Vocational Education degree, 13% a Higher Vocational Education degree, and 7% had a university degree. Thirty-three percent had completed high school and 3% had only finished elementary school.

Families were recruited as part of a study evaluating the effectiveness of PMTO in The Netherlands, in which this intervention was compared to care as usual (Thijssen et al. 2017). For obvious reasons, only families who received PMTO were used in the present study. To be included, the child had to reveal a *T*-score of 60 or higher on the externalizing subscale of the Child Behavior Checklist (CBCL; Achenbach 1991; Dutch version: Verhulst et al. 1996) and the child had to be residing at home with at least one biological or adoptive parent. Exclusion criteria were severe intellectual disability or psychopathology of the parent(s) (including substance use disorders) that would interfere with participation in treatment, sexual abuse in the family, an IQ of the child lower than 70. These exclusion criteria were assessed during intake. Parents were asked whether they were capable to attend weekly therapy sessions. When there was a suspicion of severe substance use problems or sexual abuse, this was further investigated.

Parents in the present study received PMTO from 25 certified therapists whose treatment fidelity scores were determined prior to the start of the treatment. All therapists had completed the full PMTO training program of approximately 24 months. During this training period, therapists had to treat at least three families with PMTO before they were allowed to take part in the official PMTO certification procedure. This procedure involved treating another family with PMTO. On the basis of videotaped sessions of this therapy, it was determined whether or not the therapists received their license to carry out PMTO in clinical practice. Following the completion of the training program, therapists were regularly monitored on their treatment fidelity, leading to annual recertification of their license.

### Procedure

Families were included in the period between June 2009 and January 2013. As soon as families were referred to the child service agency, it was checked whether they met the criteria for being included in the study. Families who met the criteria received information about the study and its procedure and were invited to participate. When parents agreed, they were asked to give their written consent. The study protocol was approved by the Medical Ethics Committee of Maastricht University Medical Centre.

Families were assessed at four time points: a baseline assessment (T0) and three follow-up measurements, after 6 (T1), 12 (T2), and 18 months (T3). Trained research assistants, who were not the family’s therapist, administered the interview for measuring parenting practices. A web-based system was used for the self-report assessments, which enabled parents to complete questionnaires on a computer at home. In all cases, the main caregiver (i.e., the parent who spent most time with the child) filled out the scales. All participating parents received a small financial compensation in gift vouchers for the three follow-up measurements (i.e., €10 at T1; €20 at T2; and €30 at T3).

In PMTO, the therapist works with the parent(s) of one family. The children are not present during these sessions. Role play is an important mechanism in the PMTO sessions to teach and practice the effective parenting practices. In between sessions, the therapist calls the parent to ask about homework, to provide advice and support, and to answer questions. Treatment duration depends on the family’s needs and progress throughout the therapy, but typically lasts between 15 and 25 weekly sessions.

### Measures

#### Treatment fidelity

As part of the official certification process, videotapes of four therapy sessions, were assessed by means of the FIMP rating system (Knutson et al. 2009). This rating system measures the degree of therapists’ adherence to the PMTO treatment model along five dimensions: PMTO knowledge, Structure, Teaching, Process skills, and Overall development, which will be described briefly hereafter. Each dimension is scored on a 9-point scale, in which 1–3 can be considered as “unacceptable” (“needs work”), 4–6 as “acceptable”, and 7–9 as “good”. The FIMP ratings were performed by a group of certified FIMP coders (all PMTO educators), both from the US and the Netherlands. All of the FIMP raters have passed the annual reliability tests conducted by the Oregon Social Learning Center. Each session of the same PMTO therapist was rated by a different FIMP coder. In order to ‘pass’, an average rating of 6.0 or higher (as a mean score including all 5 dimensions) was needed. In the present study, the range of FIMP scores was 5.65 to 8.20, with a mean fidelity score of 7.07.

The FIMP dimensions are defined as follows. *PMTO knowledge*, which reflects the therapist’s understanding of PMTO and SIL principles. *Structure* refers to balancing several activities during the session, such as following an agenda, maintaining an orderly flow, leading without dominating, responsiveness to family issues, and using sensitive timing and pacing. *Teaching* comprises proficiency in strategies and tools that promote parents’ mastery and use of PMTO practices. The use of verbal teaching (e.g., giving information, making suggestions, providing rationale) and active teaching (engaging the family in the learning process by brainstorming, prompting, role play, asking solution-eliciting questions) should be balanced. *Process skills* provide support to create a safe and supportive learning context (e.g., maintaining appropriate balance, encouraging skill development, joining the family’s storyline). And finally, *Overall development* reflects the accomplishment of session goals and includes, for example, the likelihood that the parents will use the procedures, the parent’s apparent satisfaction, and the likelihood they will continue with the intervention.

#### Externalizing behavior problems

The *Child Behavior Checklist* (CBCL) is a widely used rating scales for assessing behavioral and emotional problems of children aged 6 to 18 years (Achenbach 1991; Dutch version: Verhulst et al. 1996). Each scale consists of 120 items scored on a 3-point Likert scale (0 = not true; 1 = somewhat or sometimes true; 2 = very or often true). For the present study, only the externalizing scale was used. The CBCL has good reliability and validity (Achenbach 1991; Verhulst et al. 1996).

The *Parent Daily Report* (PDR) is a reliable 34-item checklist of problem behaviors (Patterson et al. 1982) and is administered by a research assistant other than the therapist. At first, the checklist is administered face-to-face with the main caregiver to assess whether specific problem behaviors of the child have occurred during the past 6 months. Next, the items to which the parent responds affirmatively are again administered via telephone on three consecutive days to examine whether these behaviors have occurred during the past 24 h. The items involve both severe (e.g., arson, stealing) and less serious problem behavior (e.g., being rude, complaining). The scores of the three consecutive days are summed to obtain the total PDR score.

#### Parenting practices

The Dutch translation of the *Caregiver Wish List* (CWL; Hodges 2005; Hodges et al. 2009) is an interview-based instrument consisting of 53 items questioning the parent about his/her parenting skills. The interviewer reads the questions to the parent, who has to indicate the most applicable response option using a 5-point Likert scale. Items are allocated to six domains of parenting skills: providing direction and following up (4 items), encouraging good behavior (5 items), discouraging undesirable behavior (24 items), monitoring activities (13 items), connecting positively with the child (3 items), and problem solving orientation (4 items). Each domain score should be regarded as a dimension with weak parenting skills on one end and strong parenting skills on the other. In the current study, only the discouraging undesirable behavior domain and the CWL total score were used, since these were the only scales showing adequate internal consistency (.80 and .76, respectively).

#### Parental stress and psychopathology

The *Nijmeegse Ouderlijke Stress Index* (NOSI; de Brock et al. 1992) is an adaptation of the Parenting Stress Index (Abidin 1983), and measures stress experienced by parents in the relationship with their child. The NOSI comprises 123 items that have to be rated on a 5-point Likert scale (1 = strongly disagree, 5 = strongly agree). Ratings on all items can be summed to create a total stress score, with higher scores reflecting higher levels of perceived stress by the parent. The NOSI has adequate reliability and validity (de Brock et al. 1992).

Psychopathological symptoms of the parents were measured by the Dutch version of the *Symptom Checklist-90 Revised* (Arrindell and Ettema 2003). The 90 items are rated using a 5-point scale (1 = no problem to 5 = very serious) to indicate the extent to which the parent has experienced the listed symptom during the previous week. In contrast to the original version of the SCL-90-R, the Dutch version comprises eight instead of nine subscales (e.g., Anxiety, Somatic Symptoms, Depression). For the present study, the SCL-90-R total score was used, for which higher scores on the SCL-90-R indicate more serious psychopathology.

#### Working alliance

The short form of the *Working Alliance Inventory* (WAI-S; Tracey and Kokotovic 1989) was used to assess the quality of the parent-therapist alliance. The WAI-S comprises 12 items that can be allocated to three subscales of four items each: (a) agreement between parent and therapist on the goals of the therapy; (b) the degree to which the tasks of the therapy will actually address the parent’s problems; and (c) the quality of the bond between the parent and the therapist. Normally, the items of the WAI-S are rated using a 7-point Likert scale. However, in the present study, a 5-point scale was used for practical reasons. Ten items are positively worded and two items are negatively worded. The scores on the negatively stated items are reversed, so that all scores can be summed to obtain a total score, with higher scores reflecting a better parent-therapist working alliance.

#### Treatment completion

The PMTO treatment was considered completed when the core of PMTO parenting skills was dealt with in a satisfactory way (good directions—encouragement—setting limits). This was assessed based on registration in the PMTO database. When therapists discussed parents’ experiences with applying the time out or work chores in multiple sessions, parents were assumed to have enough practice to set limits effectively. Of the 86 PMTO families in the present study, 64 families completed the PMTO treatment.

### Data Analyses

The proportion of missing values in this data set ranged from 0% in the demographic variables to approximately 50% on the NOSI. We applied an intention-to-treat procedure by conducting multiple imputation (Rubin 1987) to handle the missing data through a chain of conditional regression models (fully conditional specification; Van Buuren et al. 1999). We used predictive mean matching (PMM; Little 1988; Rubin 1986) for the scale variables and polytomous regression for ordered categorical data. All computations were carried out with Mice (Van Buuren and Groothuis-Oudshoorn 2011) in R (R Core Team 2014), with 150 iterations for the algorithm to converge and 25 multiply imputed datasets, using available and custom imputation routines. The outcomes over the 25 datasets were combined into a single inference using Rubin’s (1987) rules.

To examine the association between the FIMP scores and treatment outcome, Pearson correlation coefficients were calculated for the five FIMP dimensions and the FIMP total score with the outcome scores for the questionnaires measuring externalizing behavior problems, parenting skills, parenting stress, psychopathological symptoms of the parents, and working alliance. Only those measures were used for which the effectiveness study revealed significant effects of PMTO (see Thijssen et al. 2017). Treatment effects were operationalized in terms of difference scores and calculated for each assessment instrument: scores at baseline (T0) were subtracted from scores at 6 (T1), 12 (T2), and 18 (T3) months. Negative difference scores indicated improvement for externalizing behavior problems, parenting stress, and parental psychopathology. For parenting practices, improvement was indicated by positive difference scores.

## Results

First, we analyzed whether treatment fidelity was related to treatment outcome in terms of externalizing behavior problems. We calculated Pearson correlation coefficients between the five FIMP dimensions and mean FIMP total score and difference scores obtained with the CBCL and PDR at the various time points. As can be seen in Table 1, a number of significant associations emerged for the CBCL. For the T3-T0 difference score, significant negative correlations were found for three of the five FIMP dimensions: PMTO knowledge, Teaching, and Overall development. This finding indicates that the higher the score on these FIMP dimensions during therapist training, the larger the decrease in CBCL externalizing behavior from baseline to 18-months follow-up. Furthermore, a significant negative correlation was found between PMTO knowledge and the CBCL T1-T0 difference score. For the T2-T0 difference score, none of the correlations attained statistical significance and this was also the case for correlations involving PDR difference scores.

**Table 1:** Pearson correlation coefficients between FIMP scores and treatment outcome as indexed by CBCL and PDR difference scores

	CBCL			PDR		
	T1-T0	T2-T0	T3-T0	T1-T0	T2-T0	T3-T0
PMTO knowledge	−.22*	−.09	−.33**	.06	.16	.08
Structure	−.17	−.00	−.19	−.07	.06	.01
Teaching	−.12	−.03	−.23*	−.05	.04	−.01
Process skills	−.16	−.04	−.15	−.06	.05	−.01
Overall development	−.17	−.07	−.27*	.02	.12	.04
Mean total score	−.18	−.05	−.26	−.02	.09	.03

*FIMP* Fidelity of IMPlementation, *CBCL* Child Behavior Checklist, *PDR* Parent Daily Report

*p < .05 (1-tailed)

**p < .01 (1-tailed)

Second, we examined the association between treatment fidelity and changes in parenting practices. Pearson correlation coefficients were calculated between FIMP scores and the difference scores on CWL Discouraging undesirable behavior domain and the CWL total score. Table 2 shows that only one correlation reached significance for Discouraging undesirable behavior: that is, FIMP Overall development was positively associated with discouraging undesirable behavior from T0 to T3, indicating that the higher the score on Overall development, the more improvement parents reported on Discouraging undesirable behavior. For the CWL total score, both the T1-T0 and T3-T0 difference scores showed significant positive correlations for the FIMP dimensions referring to Structure, Teaching, and Overall development. These results show that the higher a PMTO therapist’s scores on these FIMP dimensions, the greater the improvement in parenting skills. Again, no significant correlations were found for the T2-T0 difference score.

**Table 2:** Pearson correlation coefficients between FIMP scores and treatment outcome as indexed by CWL discouraging undesirable behavior domain and CWL total difference scores

	Discouraging undesirable behavior			CWL total score		
	T1-T0	T2-T0	T3-T0	T1-T0	T2-T0	T3-T0
PMTO knowledge	−.07	−.10	.06	.11	−.06	.09
Structure	.05	.05	.14	.27*	.06	.21*
Teaching	.04	.08	.18	.21*	.10	.24*
Process skills	.01	.02	.10	.16	.03	.15
Overall development	.07	.09	.21*	.24*	.12	.26*
Mean total score	.02	.03	.15	.22	.05	.21

*FIMP* Fidelity of IMPlementation, *CWL* Caregiver Wish List

*p < .05 (1-tailed)

Third, we examined the association between treatment fidelity and parental psychopathology. Difference scores of the NOSI and SCL-90-R were correlated with the FIMP dimensions. As shown in Table 3, for the NOSI, a significant negative correlation was found between the T1-T0 difference score and FIMP Process skills. Furthermore, all FIMP dimensions were significantly negatively associated with the NOSI T3-T0 difference score. For the SCL-90-R, a significant correlation was found between the T3-T0 difference score and FIMP PMTO knowledge. Thus, a higher score on this dimension is related to a larger decrease in psychopathological symptoms reported by parents. No significant correlations were found for the T1-T0 and T2-T0 difference scores in parental psychopathology.

**Table 3:** Pearson correlation coefficients between FIMP scores and treatment outcome as indexed by NOSI and SCL-90-R difference scores

	NOSI			SCL-90-R		
	T1-T0	T2-T0	T3-T0	T1-T0	T2-T0	T3-T0
PMTO knowledge	−.17	−.11	−.30*	−.13	−.10	−.23*
Structure	−.23	−.11	−.30*	−.11	−.13	−.21
Teaching	−.18	−.12	−.28*	−.07	−.10	−.15
Process skills	−.26*	−.13	−.30**	−.17	−.12	−.18
Overall development	−.21	−.16	−.31**	−.08	−.13	−.20
Mean total score	−.22	−.13	−.32*	−.12	−.13	−.21

*FIMP* Fidelity of IMPlementation, *NOSI* Parenting Stress Index, *SCL-90-R* Symptom Check List-90-Revised

*p < .05 (1-tailed)

**p *<* .01 (1-tailed)

Fourth, we tested whether FIMP scores were related to working alliance. Scores on the WAI-S at T1, T2, and T3 were used in the analyses. No significant correlations were found between the FIMP dimensions and the WAI-S scores, implying that FIMP scores were unrelated to the alliance between therapist and parents.

Finally, we examined whether FIMP scores were related to treatment completion. Independent samples *t*-tests were performed to compare completers and drop-outs on the five FIMP dimensions. Significant differences between treatment completers and drop-outs were documented for all FIMP dimensions (see Table 4). Therapists’ of families who completed the core of the PMTO treatment had mean FIMP scores in the ‘good work’ range on all dimensions, whereas therapists’ of families who dropped out had scores in the “acceptable” range. This indicates that parents were more likely to complete the PMTO treatment when their therapists had higher fidelity ratings at certification as PMTO therapists.

**Table 4:** Mean FIMP scores of treatment completers and drop-outs

	completers (*n* = 64)	drop-outs (*n* = 22)	*t*	*p*
PMTO knowledge	7.10	6.66	2.70	.01
Structure	7.23	6.55	4.19	.00
Teaching	7.03	6.31	4.20	.00
Process skills	7.43	7.02	3.67	.00
Overall development	7.23	6.70	4.09	.00
Mean total score	7.20	6.65	4.12	.00

The maximum score is 9. *FIMP* Fidelity of Implementation

## Discussion

The aim of the present study was to examine whether treatment fidelity scores obtained for PMTO certification purposes prior to the intervention would be associated with treatment completion and with larger treatment effects on various outcome variables, including child externalizing behavior problems, parenting practices, parental psychopathology, parenting stress, working alliance. This study explored the link between treatment fidelity during therapist training and multiple outcome measures of PMTO. Previous studies on this topic mainly focused on the association between FIMP scores and parenting practices or externalizing behavior problems as the outcome variable (Forgatch and DeGarmo 2011; Forgatch et al. 2005; Hukkelberg and Ogden 2013).

In line with earlier investigations, the results of the present study provide indications that treatment fidelity is indeed related to greater improvements in child externalizing behavior. The higher therapists’ level of PMTO knowledge the larger the change in externalizing behavior on the CBCL between baseline and 6 months. Furthermore, higher levels of PMTO knowledge, Teaching, and Overall development were related to larger improvements in externalizing behavior at the 18-months follow-up. This points out that higher treatment fidelity scores of the therapist on these dimensions were accompanied by a larger decrease in externalizing behavior on the CBCL, and that this was especially the case at the 18-months follow-up. It is noteworthy that the 18-months follow-up point lies well beyond the PMTO treatment period, which usually lasts between 6 and 9 months. Thus, our findings point to the relevance of treatment fidelity of PMTO’s longer term effectiveness. Remarkably, no significant associations were found between FIMP scores and PDR difference scores. However, the treatment effect based on the PDR (*d* = .32) is lower compared to the treatment effect based on the CBCL (*d* = .71; Thijssen et al. 2017). This could explain why no associations were found between the FIMP and PDR scores.

With regard to parenting practices, it was found that higher scores on the FIMP dimension Overall development were related to larger increases in the use of discouraging undesirable behavior from baseline to 18-months follow-up. Higher CWL total scores were associated with higher levels of the FIMP dimensions Structure, Teaching, and Overall development from baseline to 6 and 18 months. Our findings imply that higher treatment fidelity is associated with larger improvements in parenting practices and with an increase in discouragement of undesirable behavior in particular. Treatment fidelity was also related to decreases in parenting stress and parental psychopathology. Parents experienced significantly less parenting stress after 6 months when therapists had higher levels of Process skills. After 18 months, higher scores on all FIMP dimensions were associated with decreased levels of parenting stress. Psychopathological symptoms were significantly lower after 18 months when therapists had higher levels of the FIMP dimension PMTO knowledge. Thus, parents report less parenting-related stress and psychopathological symptoms, especially at 18-months follow-up, when their therapists more strongly adhere to the PMTO program. Meanwhile, treatment fidelity was not associated with greater working alliance, in other words, parents were not more satisfied with their therapist and the intervention when their therapist adhered more strictly to PMTO guidelines. Working alliance appears independent of how well the therapist delivers the PMTO treatment.

Most significant associations were found from baseline to 18-months follow-up, whereas fewer significant associations could be documented from baseline to 6 months and from baseline to 12 months. This suggests that high treatment fidelity during PMTO therapist training is particularly important for the long-term effects of PMTO. For the endurance of the effects of PMTO, it seems to be essential that the therapist strongly adheres to the PMTO method. Note that in the present study all therapists had been certified and therefore all of them had been judged as sufficiently adherent. To achieve optimal long-term treatment effects, it seems to be necessary for PMTO therapists to perform better than merely sufficient.

The FIMP dimensions Structure and Overall development were most consistently related to treatment outcome measures and, thus, seem to be the most important dimensions of treatment fidelity. Hence, the therapist should be especially capable of balancing several activities during the session, to increase the likelihood that the parents apply the PMTO parenting strategies, and enabling them to manage unique or difficult situations. PMTO knowledge was mainly related to a decrease in externalizing behavior problems, suggesting that the therapist’s understanding of PMTO and the SIL principles are important for improvement in externalizing behavior problems, while creating balance between several activities during the session seems to be important for improvements in parenting practices. The FIMP Process skills dimension was only related to a decrease in parenting stress. Therefore, creating a safe and supportive learning context seems to be important in reducing stress of parents, which might enable them to benefit more from the treatment.

Finally, we examined whether FIMP scores differed between therapists of families who completed the treatment and families who dropped out. Parents who completed PMTO more often had a therapist who adhered more strongly to the PMTO principles than parents who dropped out. This implies that PMTO therapists scoring higher on treatment fidelity at certification are better able to keep parents in treatment than therapists showing lower treatment fidelity.

Previous studies into PMTO treatment fidelity showed that competent adherence to the PMTO principles predicted the degree of change in parenting (i.e., Forgatch and DeGarmo 2011; Forgatch et al. 2005; Hukkelberg and Ogden 2013). These previous studies used a different analytical approach, which makes direct comparison with our results difficult. However, two studies reported descriptive statistics, including correlations. In the study of Hukkelberg and Ogden (2013), none of the correlations between the FIMP total score and child problem behavior outcomes were significant. Forgatch and DeGarmo (2011) only found a significant positive correlation between the mean FIMP total score and fathers’ post-treatment parenting (*r* = .27). FIMP scores were not significantly related to change in mothers’ and fathers’ parenting. The differences in correlations between the present study and previous studies could be explained by the different approach of the studies. The present study examined more aspects of treatment fidelity than previous studies on treatment fidelity in PMTO. None of the previous studies examined the separate FIMP dimensions. Our study showed that some dimensions are more strongly related to therapy outcome variables than others, which could be useful information for improving the treatment method. Further, in contrast to the previous studies on treatment fidelity in PMTO, the present study investigated the relations between fidelity during training and effectiveness at subsequent assessment points and showed that treatment fidelity was especially related to longer-term outcomes.

Some limitations of the present study should be mentioned. First, the present study was correlational in nature. Obviously, this type of research indicates that there is a relationship between two variables, but cannot prove that one variable causes a change in the other. Second, therapists’ FIMP scores obtained during certification were used; these scores pertained to other families than the families treated in the present study. It is possible that some therapists deteriorated in terms of fidelity while others improved since the time of their certification. As a result, PMTO therapists might have performed differently during certification than during treatment of the PMTO families in the present study. However, correlations between the FIMP scores and outcome measures found in our study are not lower than those reported in previous studies in which FIMP scores were obtained from the same families as the outcome variables (see Forgatch and DeGarmo 2011; Hukkelberg and Ogden 2013). Furthermore, Hukkelberg and Ogden (2013) found that PMTO therapists’ fidelity scores were relatively stable over a period of approximately 9 months (correlations ≥ .30). Therefore, FIMP scores obtained prior to treatment seem to be appropriate as a proxy measure of subsequent treatment fidelity.

In conclusion, the current study found support for the notion that treatment fidelity during PMTO training is related to later treatment outcome. The higher the therapist’s fidelity scores at certification, the larger the improvements in externalizing behaviors, parenting practices, parenting stress, and parental psychopathological symptoms. This was not translated into higher levels of working alliance; parents were not more satisfied with their therapist and the intervention when the therapist was more adherent to the protocol. The finding that treatment fidelity was most consistently associated with treatment outcome at the 18-months follow-up assessment indicates that for the long-term effects of PMTO, it is important that PMTO is delivered as intended by the developers and according to the treatment principles in more than merely an acceptable fashion. Furthermore, it was found that parents were more likely to complete the PMTO treatment when their therapist strongly adheres to the PMTO principles. Based on these results, it seems advisable to raise the bar for certification, because performing acceptably seems to be insufficient for attaining positive long-term treatment effects with PMTO.
